# Supplementary material for: Gold Nanoparticles-Functionalized Ultrathin Graphitic Carbon Nitride Nanosheets for Boosting Solar Hydrogen Production: The Role of Plasmon-Induced Interfacial Electric Fields
Source: Molecules. 2025 Aug 18;30(16):3406. doi: 10.3390/molecules30163406 (PMC12388607; doi:10.3390/molecules30163406)
Supplement: Supplementary file 1 [file molecules-30-03406-s001.zip › molecules-3773055-supplementary.pdf]

Supporting information

Gold Nanoparticles-Functionalized Ultrathin Graphitic Carbon Nitride Nanosheets for Boosting Solar Hydrogen Production: The Role of Plasmon-Induced Interfacial Electric Fields

Haidong Yu [1], Ziqi Wei [1], Qiyue Gao [1], Ping Qu [2], Rui Wang [1], Xuehui Luo [1], Xiao Sun [1], Dong Li [1], Xiao Zhang [1], Jiufen Liu [3,*] and Liang Feng [1,*]

[1] Langfang Natural Resources Comprehensive Survey Center, China Geological Survey, Langfang 065000, China; yuhaidong1992@163.com (H.Y.); 13115333803@163.com (Z.W.); gqy7770119@163.com (Q.G.); wrui@mail.cgs.gov.cn (R.W.); ls19760811@163.com (X.L.); sunxiao@mail.cgs.gov.cn (X.S.); 17852033981@163.com (D.L.); xiao.zhang@cug.edu.cn (X.Z.)

[2] Center for Geophysical Survey, China Geological Survey, Langfang 065000, China; quping_cgs@163.com

[3] Natural Resources Comprehensive Survey Command Center, China Geological Survey, Beijing 100055, China

* Correspondence: 13863858360@163.com (J.L.); fengliang@mail.cgs.gov.cn (L.F.)

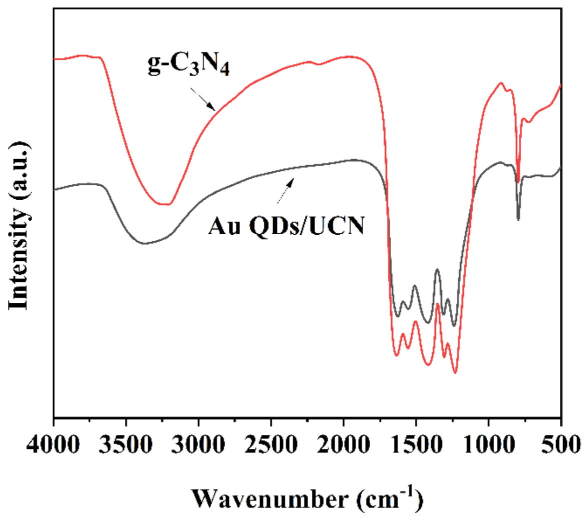

Figure S1 FT-IR spectra of Au QDs/UCN and g-C_3N_4.

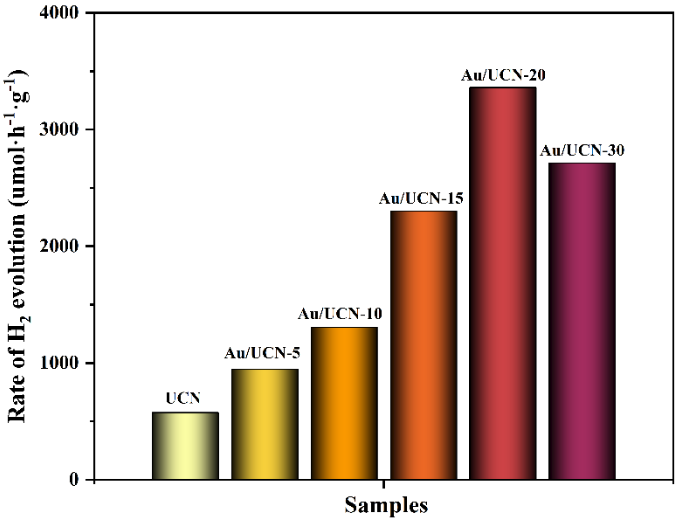

Figure S2 Hydrogen evolution rates of UCN and Au/UCN photocatalysts.

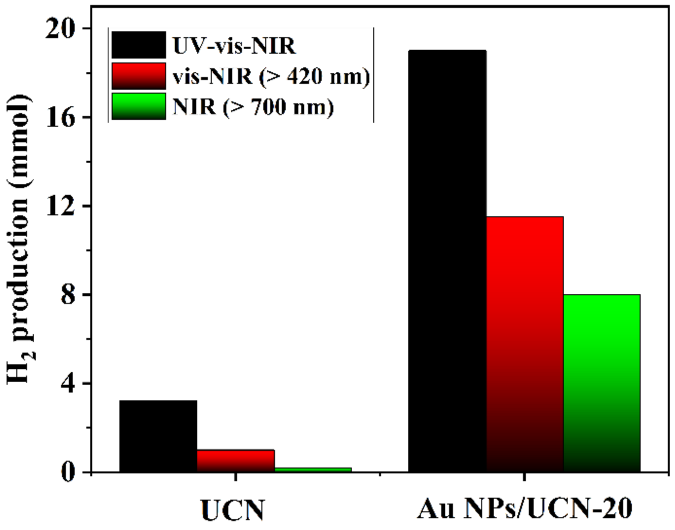

Figure S3 Comparison of H_2 production rate over UCN and Au NPs/UCN-20 samples under UV-vis-NIR, vis-NIR and NIR light irradiation.

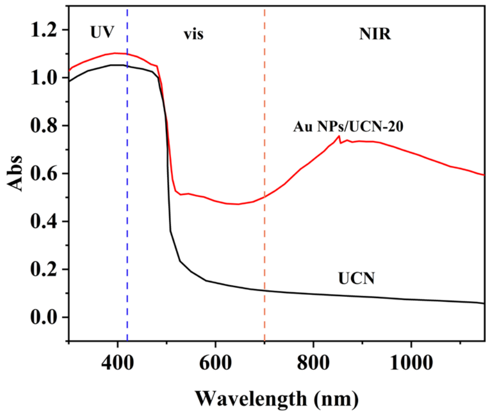

Figure S4 The light absorption characteristics of UCN and Au NPs/UCN-20 (UV, vis, NIR).

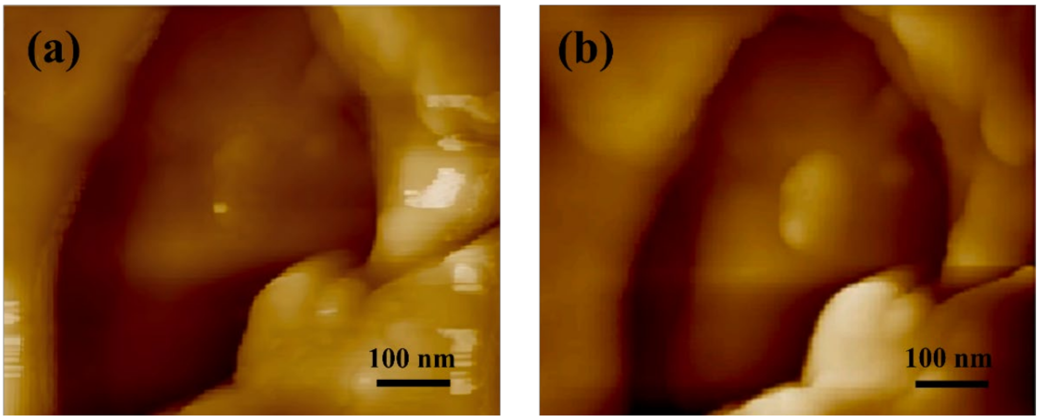

Figure S5 AFM of Au QDs/UCN before (a) and after (b)illumination.

Table S1. The amount of reagent added for preparing Au/UCN with different content of Au (Determine the actual content of gold through ICP-MS verification).

Sample	$g\text{-}C_3N_4$/g	$HAuCl_4{\cdot}4H_2O$(0.01M)/mL	Au (%) Theoretical	Au (%) Actual
UCN	0.4	0	0	0
Au/UCN-5	0.4	5	2.40 %	1.31 %
Au/UCN-10	0.4	10	4.69 %	2.26 %
Au/UCN-15	0.4	15	6.88 %	2.89 %
Au/UCN-20	0.4	20	8.97 %	3.97 %
Au/UCN-30	0.4	30	12.87 %	5.44 %
